# Dopamine transporter deficiency syndrome: phenotypic spectrum from infancy to adulthood

**DOI:** 10.1093/brain/awu022

**Published:** 2014-03-10

**Authors:** Joanne Ng, Juan Zhen, Esther Meyer, Kevin Erreger, Yan Li, Naseebullah Kakar, Jamil Ahmad, Holger Thiele, Christian Kubisch, Nicholas L. Rider, D. Holmes Morton, Kevin A. Strauss, Erik G. Puffenberger, Daniela D’Agnano, Yair Anikster, Claudia Carducci, Keith Hyland, Michael Rotstein, Vincenzo Leuzzi, Guntram Borck, Maarten E. A. Reith, Manju A. Kurian

**Affiliations:** 1 Neurosciences Unit, UCL Institute of Child Health, London, UK; 2 Department of Neurology, Great Ormond Street Hospital, London, UK; 3 Department of Psychiatry, New York University School of Medicine, New York, USA; 4 Department of Molecular Physiology and Biophysics, Vanderbilt University, Nashville, USA; 5 Institute of Human Genetics, University of Ulm, Ulm, Germany; 6 Department of Biotechnology and Informatics, BUITEMS, Quetta, Pakistan; 7 Cologne Centre for Genomics (CCG), University of Cologne, Cologne, Germany; 8 Medical Arts Allergy, Carlisle, Pennsylvania, USA; 9 Clinic for Special Children, Strasburg, Pennsylvania, USA; 10 Department of Biology and Biological Foundations of Behaviour Program, Franklin & Marshall College, Lancaster, Pennsylvania, USA; 11 Lancaster General Hospital, Lancaster, Pennsylvania, USA; 12 Department of Paediatrics, Child Neurology and Psychiatry, Sapienza, University of Rome, Rome, Italy; 13 Metabolic Diseases Unit, Edmond and Lily Safra Children’s Hospital, Tel Hashomer, Israel; 14 Department of Experimental Medicine, Sapienza, University of Rome, Rome, Italy; 15 Medical Neurogenetics, LLC, Atlanta, Georgia, USA; 16 Department of Paediatric Neurology, The Tel Aviv Sourasky Medical Centre, Tel Aviv, Israel; 17 Department of Biochemistry and Molecular Pharmacology, New York University School of Medicine, New York, USA

**Keywords:** dopamine, dopamine transporter (DAT), juvenile, parkinsonism, dystonia, *SLC6A3*

## Abstract

Dopamine transporter deficiency syndrome due to *SLC6A3* mutations is the first inherited dopamine ‘transportopathy’ to be described, with a classical presentation of early infantile-onset progressive parkinsonism dystonia. In this study we have identified a new cohort of patients with dopamine transporter deficiency syndrome, including, most significantly, atypical presentation later in childhood with a milder disease course. We report the detailed clinical features, molecular genetic findings and *in vitro* functional investigations undertaken for adult and paediatric cases. Patients presenting with parkinsonism dystonia or a neurotransmitter profile characteristic of dopamine transporter deficiency syndrome were recruited for study. *SLC6A3* mutational analysis was undertaken in all patients. The functional consequences of missense variants on the dopamine transporter were evaluated by determining the effect of mutant dopamine transporter on dopamine uptake, protein expression and amphetamine-mediated dopamine efflux using an *in vitro* cellular heterologous expression system. We identified eight new patients from five unrelated families with dopamine transporter deficiency syndrome. The median age at diagnosis was 13 years (range 1.5–34 years). Most significantly, the case series included three adolescent males with atypical dopamine transporter deficiency syndrome of juvenile onset (outside infancy) and progressive parkinsonism dystonia. The other five patients in the cohort presented with classical infantile-onset parkinsonism dystonia, with one surviving into adulthood (currently aged 34 years) and labelled as having ‘juvenile parkinsonism’. All eight patients harboured homozygous or compound heterozygous mutations in *SLC6A3*, of which the majority are previously unreported variants. *In vitro* studies of mutant dopamine transporter demonstrated multifaceted loss of dopamine transporter function. Impaired dopamine uptake was universally present, and more severely impacted in dopamine transporter mutants causing infantile-onset rather than juvenile-onset disease. Dopamine transporter mutants also showed diminished dopamine binding affinity, reduced cell surface transporter, loss of post-translational dopamine transporter glycosylation and failure of amphetamine-mediated dopamine efflux. Our data series expands the clinical phenotypic continuum of dopamine transporter deficiency syndrome and indicates that there is a phenotypic spectrum from infancy (early onset, rapidly progressive disease) to childhood/adolescence and adulthood (later onset, slower disease progression). Genotype–phenotype analysis in this cohort suggests that higher residual dopamine transporter activity is likely to contribute to postponing disease presentation in these later-onset adult cases. Dopamine transporter deficiency syndrome remains under-recognized and our data highlights that dopamine transporter deficiency syndrome should be considered as a differential diagnosis for both infantile- and juvenile-onset movement disorders, including cerebral palsy and juvenile parkinsonism.

## Abstract (precis)

Dopamine transporter deficiency syndrome is an *SLC6A3*-related progressive infantile-onset parkinsonism-dystonia that mimics cerebral palsy. *Ng et al*. describe clinical features and molecular findings in a new cohort of patients. They report infants with classical disease, as well as young adults manifesting as atypical juvenile-onset parkinsonism-dystonia, thereby expanding the disease spectrum.

## Introduction

Dopamine dyshomeostasis is associated with several neurological and neuropsychiatric conditions including Parkinson’s disease, depression, schizophrenia, attention deficit hyperactivity disorder, autism and drug addiction (Carlsson, 1987; Greengard, 2001; Adinoff, 2004; Mehler-Wex *et al.*, 2006; Mazei-Robison *et al.*, 2008). A key step in the duration and intensity of dopamine signalling is the reuptake of extracellular dopamine that is principally mediated by the presynaptic dopamine transporter (DAT), a Na^+^/Cl^−^-dependent neurotransmitter sodium symporter, expressed by dopaminergic neurons (Torres *et al.*, 2003*a*). Recently, we have described loss-of-function mutations in the gene encoding DAT (*SLC6A3*) in a clinical syndrome of autosomal recessive infantile parkinsonism dystonia with raised dopamine metabolites in CSF (Assmann *et al.*, 2004; Kurian *et al.*, 2009, 2011*a*). The classical dopamine transporter deficiency syndrome (DTDS) phenotype is characterized by an infantile-onset hyperkinetic movement disorder with progression to severe parkinsonism during early childhood (Kurian *et al.*, 2009, 2011*a*). In this new study we describe a unique cohort of patients with DTDS and expand the phenotypic spectrum and disease continuum to include adolescents and young adults with atypical disease features.

## Materials and methods

### Clinical cases

Patients presenting with childhood onset parkinsonism dystonia and, where available, a neurotransmitter profile characteristic for DTDS [raised CSF homovanillic acid: 5-hydroxyindoleacetic acid (HVA: 5-HIAA) ratio >4.0] (Kurian *et al.*, 2009, 2011*a*) were identified through international contact with child neurologists with specialist expertise in movement disorders and clinical geneticists. Referring clinicians contacted M.A.K directly to see whether the clinical and biochemical features of their patients were suggestive of DTDS. Thus, eight such patients were identified for *SLC6A3* analysis and inclusion in this study. Each patient’s clinical case notes were reviewed in detail to determine: (i) the clinical features at presentation; (ii) results of neurological investigations including CSF neurotransmitters; (iii) disease course; (iv) response to medication; and (v) long-term clinical outcome. All patients underwent neurological examination and video footage of the movement disorder was undertaken with written informed consent obtained from participant families. Three cases did not consent to video recording because of cultural beliefs but were thoroughly clinically assessed to document movement disorder and neurological features. Five cases consented to video recording and a detailed video was taken to demonstrate the general phenotype, gross motor features at rest, fine motor tasks and eye movements. Four child neurologists with specialist interest in movement disorders (J.N., V.L., M.R., M.A.K.) reviewed the videos available to obtain consensus opinion. Videos on Cases 1–3 and 6 are available in the Supplementary material.

### *SLC6A3* mutational analysis

For Cases 1–3 (three siblings from a consanguineous Pakistani family) we performed whole exome sequencing on one affected individual (Case 3), using a HiSeq 2000 sequencer with a paired-end 2 × 100 bp protocol after enrichment of exonic and adjacent splice site sequences with the SeqCap EZ Human Exome Library v.3.0 enrichment kit. This resulted in a mean coverage of 97;79% of target sequences were covered at least 30×. Data analysis and filtering was performed as previously described (Basel-Vanagaite *et al.*, 2012). Validation of variants detected by exome sequencing and co-segregation analysis in the family were performed by Sanger sequencing of PCR products on an ABI 3730 DNA Analyzer using BigDye® chemistry v3.1. The novelty of all identified variants was determined by comparison with established variant databases (including dbSNP137, 1000 Genomes SNP calls and the NHLBI Exome Sequencing project) as well as direct sequencing of ethnically-matched 150–300 control chromosomes. For Cases 4–8, direct Sanger sequencing of *SLC6A3* was undertaken using gene primers covering all coding exons and flanking intronic regions.

### *In vitro* heterologous expression system

*In vitro* functional studies of identified missense mutations (Ala314Val, Gly386Arg, Tyr470Ser, Arg85Leu, Arg445Cys, co-expressed Arg85Leu–Arg445Cys) were performed after preparation of mutant constructs of human DAT from wild-type pCIN4-hDAT, as previously described (primers available on request) (Kurian *et al.*, 2009, 2011*a*; Stouffer *et al.*, 2011). Culturing and transient transfection of LLC-PK_1_ cells with mutant and wild-type human DAT was performed by use of Lipofectamine® 2000 (Invitrogen) as previously described (Kurian *et al.*, 2009, 2011*a*). Uptake of ^3^H-dopamine (10 nM final concentration, 48 Ci/mmol, Perkin Elmer) into suspended cells expressing human DAT was measured for 5 min at 21°C, and to monitor cocaine analogue binding, cells were incubated with 4 nM ^3^H-CFT (2β-carbomethoxy-3β-[4-fluorophenyl]-tropane, 85.9 Ci/mmol, Perkin Elmer) for 20 min at 21°C; for saturation analysis, 0.1–100 nM non-radioactive CFT was also present. Briefly, uptake and binding assays used a high sodium, low potassium buffer containing glucose and tropolone, and the non-specific binding was defined with 1 μM CFT as in our previous work. (Kurian *et al.*, 2009, 2011*a*). In one set of experiments the K_m_ and V_max_ of ^3^H-dopamine was monitored in intact attached LLC-PK_1_ cells as described previously (Stouffer *et al.*, 2011). The binding affinity (Kd) and the maximum binding of ^3^H-CFT were calculated with non-linear regression by use of Radlig software (KELL program). Half maximal inhibitory concentration was estimated by logistic fitting of data by the ORIGIN software (Origin Lab Co.); this value was then entered into the Cheng- Prusoff equation to calculate the potency (K_i_) of dopamine in inhibition of ^3^H-CFT binding. K_m_ and V_max_ values for uptake were calculated with the KELL program as in previous work (Li *et al.*, 2004).

### Immunoblotting studies

Cells transiently expressing wild-type or mutant human DAT were washed with cold PBS and incubated with sulpho-NHS-SS-biotin (1 mg/ml PBS; Pierce Biotechnology) for 60 min at 4°C, before incubation with 100 mM glycine in PBS for 20 min and extensive washing. The washed cells were lysed in mammalian protein extraction reagents (Thermo Scientific) supplemented with a protease inhibitor cocktail (Thermo Scientific) for 10 min at 21°C and transferred into eppendorf vials. The vials were incubated for 60 min on ice with vortexing every 5 min. The lysate was then centrifuged at 14 000 *g* for 15 min at 4°C and supernatant was collected for preparing total lysates and separating biotinylated cell surface proteins. The biotinylated proteins were separated with immobilized monomeric NeutrAvidin (Thermo Scientific) and eluted with SDS-PAGE sample buffer. The total lysates and biotinylated proteins were resolved on 8% Tris-glycine mini gels and probed with polyclonal anti-DAT antibody against the C-terminal of DAT (Millipore), before horseradish peroxidase-conjugated goat anti-rabbit antibody. Polyclonal anti-β-actin antibody (Sigma-Aldrich) was used as an internal control for loading. The transporter signal was visualized using Thermo Scientific SuperSignal® West Pico Chemiluminescent Substrate solution (Thermo Scientific).

### Statistical analysis

Each case was assessed for transporter properties with corresponding wild-type controls. Wild-type values were combined in the overall statistical analysis of ^3^H-CFT binding data. The latter binding results for wild-type and mutant human DAT were first compared by one-way ANOVA. If this test indicated significant differences between groups, the Dunnett multiple comparisons test was used to compare each mutant value with wild-type. For statistical analysis of ^3^H-dopamine uptake by mutants, K_i_ values for wild-type human DAT (controls) were used from the same set; thus values for Cases 1–3 constituted one set and values for Case 8 another set (Case 6 and 7 were not included in this statistical analysis as there was no specific transporter activity). The mutant Ki values were expressed as % Control with Control = wild-type, set to 100%; the mutant values were then subjected to a one-sample Student’s *t*-test (Bonferroni-corrected for multiple comparisons). K_m_ and V_max_ values were obtained for one mutant and compared with wild-type values obtained in the same set by unpaired Student’s *t*-test. Indicated as *n* is the number of independent experiments (each assayed in triplicate), for which mean ± standard error (SE) was calculated. We regarded *P-*values of 0.05 or lower as statistically significant.

### Amperometry

Amperometry studies were possible for mutant Arg445Cys as there was appreciable expression on the cell surface. HEK293 cells were cultured and transiently transfected as previously described (Bowton *et al.*, 2010). Patch-clamp electrophysiology was performed using an Axopatch 200B amplifier and Clampex9 software (Molecular Devices). Amperometric measurement of dopamine was performed by a second Axopatch 200B. A 5 µm carbon fibre electrode was juxtaposed to the plasma membrane and held at +700 mV (a potential greater than the oxidation potential of dopamine). Amperometric signals were sampled at a rate of 200 Hz and low-pass filtered at 10 Hz. Traces were digitally low-pass filtered offline at 1 Hz for display only. For patch loading of dopamine, quartz recording pipettes with a resistance of 3–5 MΩ were filled with a dopamine-containing internal solution (2 mM dopamine, 110 mM KCl, 10 mM NaCl, 10 mM HEPES, 0.1 mM CaCl_2_, 2 mM MgCl_2_, 1.1 mM EGTA, 30 mM d-glucose, pH 7.35). Cells were washed with external bath solution (130 mM NaCl, 10 mM HEPES, 34 mM d-glucose, 1.5 mM CaCl_2_, 0.5 mM MgSO_4_, 1.3 mM KH_2_PO_4_, pH to 7.35). Upon gaining whole-cell access to the cell, dopamine-containing internal solution was allowed to diffuse into the cell under current clamp for 10 min before amphetamine or cocaine application.

## Results

### Clinical features of the cohort

The clinical features of all eight cases are summarized in Table 1. Our data indicates that some patients (*n = *5) presented <1 year of age (akin to previously reported children with DTDS) but there were others who presented beyond the infantile period (*n = *3).

**Table 1:** Summary of Clinical features

Family	Family 1			Family 2		Family 3	Family 4	Family 5
Case	1 (Supplementary Video 1)	2 (Supplementary Video 2)	3 (Supplementary Video 3)	4	5	6 (Supplementary Video 4 1,2)	7	8
Current age (years)	16	26	28	35	died at 10	7	4	3
Gender	M	M	M	F	F	M	M	F
Ethnicity	Pakistani	Pakistani	Pakistani	Mixed European	Mixed European	Italian	Mixed European	Mixed Ashkenazi Jew/Iranian/
								Yemeni/Turkish
Consanguinity	Yes	Yes	Yes	Yes	Yes	No	Yes	No
Presenting age (years)	11	11	11	3 months	2 months	3 months	3 months	9 months
Presenting symptoms	Tremor	Tremor	Tremor	Dystonia parkinsonism	Dystonia parkinsonism	Dystonia parkinsonism	Dystonia parkinsonism	Dystonia parkinsonism
					Dyskinetic movements	Dyskinetic movements	Dyskinetic movements	Dyskinetic movements
Age at diagnosis (years)	16 years	25 years	28 years	34 years	10 years (died)	5.6 years	3 years	1.5 years
Current symptoms								
Tremor	Titubation	Titubation	Titubation	Hand tremor	No	No	No	No
	Hand tremor	Hand tremor	Hand tremor					
Parkinsonism	No	Hypomimia	Hypomimia	Bradykinesia	No	Bradykinesia	Bradykinesia	Bradykinesia
			Bradykinesia					
			Impaired balance					
Dystonia	No	Neck	No	Yes	Limbs + axial hypotonia	Limbs + axial hypotonia	Limbs + axial hypotonia	Limbs + axial hypotonia
Generalized dyskinetic movements	No	No	No	No	Yes	Yes	Yes	Yes
						Chorea		Chorea
Eye movement	Normal	Normal	Ocular flutter	Normal	Normal	Oculogyric crisis	Normal	Saccadic intrusion on smooth pursuit
Bulbar dysfunction	No	Loss of speech to single words	Complete loss of speech	Gastrostomy	Gastrostomy	Gastrostomy	Gastrostomy	Gastrostomy
Treatments tried, (no response unless otherwise stated)	n/a	n/a	n/a	l-DOPA (initial mild)	l-DOPA	l-DOPA	Haloperidol (mild)	l-DOPA (unsustained improvement with dyskinetic movements and irritability
				Benzodiazepine (improved)	Benzodiazepine			
				Tetrabenazine	Tetrabenazine	Pergolide	Tyrosine dietaryrestriction	Selegiline (improved bradykinesia)
				Trihexyphenydyl	Trihexyphenydyl	Baclofen		
				Haloperidol	Haloperidol	Tetrabenazine		

n/a = not applicable

### Atypical dopamine transporter deficiency syndrome: Juvenile Parkinsonism Dystonia

**Figure 1:**
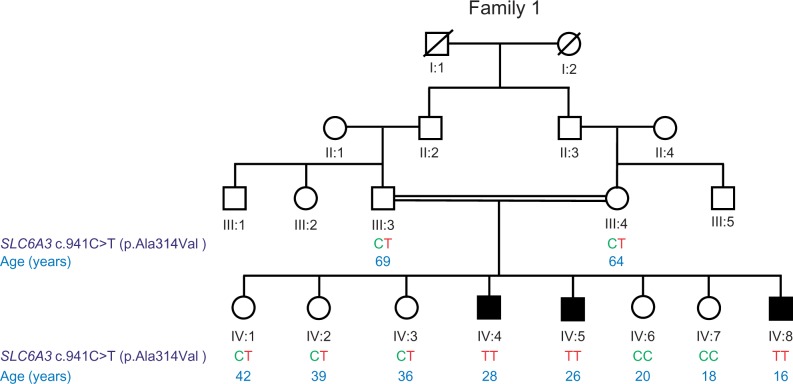
Family tree for Cases 1–3. Four generation family tree representing family members and ancestral relatives of Patients 1 (IV:8), 2 (IV:5) and 3 (IV:4). These brothers are three of eight children from Pakistani consanguineous first cousin parents. They have five healthy unaffected sisters. The parents’ (III:3 and III:4) fathers (II:2 and II:3) are brothers. Females are represented by circles and males by squares. DTDS disease status is indicated by black shading. Consanguinity is represented by a horizontal parallel double bar. Diagonal lines indicate deceased individuals (I:1 and I:2). Genotypes are indicated for *SLC6A3* mutation c.914C>T (p. Ala314Val) and indicate that parents (III:3 and III:4) are both heterozygous carriers (CT), the affected children (IV:4 IV:5 IV:6) are homozygous for the mutation (TT) and the unaffected siblings are either wild-type (CC) (IV:6 and IV:7) or heterozygous carriers (CT) (IV:1 IV:2 and IV:3).

We identified three brothers (Cases 1–3; currently aged 16, 26 and 28 years) from a Pakistani family with a clinical diagnosis of juvenile-onset parkinsonism presenting with tremor and progressive parkinsonian symptoms. The parents are consanguineous first cousins sharing common paternal grandparents (Fig. 1). There is no family history of movement disorders, Parkinson’s disease or attention deficit hyperactivity disorder or other psychiatric illness. The three brothers have five unaffected sisters aged between 18–42 years (Fig. 1).

All three brothers had a normal birth history, neonatal course, and achieved normal early developmental milestones in infancy. Age appropriate cognitive, motor and speech and language skills were reported in the first decade of life with no neurodevelopmental concerns. At 10–11 years of age, they all developed tremor affecting the head, with progression of symptoms in their 20 s, clearly observed in the elder brothers.

Currently at 16 years old, the youngest brother (IV:8; Case 1) has normal eye movements and facial expression, with predominant head tremor/titubation (Supplementary Video 1). He has normal speech, good balance and normal gait. He also reports intermittent tremor affecting hands and lower limbs. The second brother (IV:5; Case 2) now 26 years old, presented in the same manner as his younger brother with mild tremor affecting the head and hands. His movement disorder has evolved to develop cervical dystonia resulting in cervical antecollis with hypertrophy of trapezius muscles. He has frontalis hyperactivity with an associated intermittent low amplitude fine head tremor. He has normal eye movements with hypomimic facies (Supplementary Video 2). He also reports intermittent fine hand tremor and deterioration in speech and now speaks in single words only. The eldest brother is aged 28 years (IV:4; Case 3) and presented with the same symptoms as his two brothers initially. Over the past 20 years his symptoms have progressed and he is the most severely affected of the three. He now has prominent head tremor, a coarse resting hand tremor with ocular flutter and hypomimia (Supplementary Video 3). He experiences recurrent falls associated with poor balance and difficulty in initiating movements. His speech deteriorated initially to the use of single words from 20 years old onwards and now he is non-verbal. There is no evidence of cerebellar or pyramidal signs on neurological examination of any of these patients to date. Unfortunately CSF neurotransmitter studies and neuroimaging studies are not available to this family who are from a rural area of Pakistan, and medication trials with l-DOPA, dopamine agonists or others have not been undertaken.

### Classical dopamine transporter deficiency syndrome: Infantile parkinsonism dystonia

Five patients (Cases 4–8; two males) were identified with a clinical phenotype and CSF neurotransmitter profile (undertaken in 4/5 cases) compatible with DTDS. They all presented during infancy with a complex movement disorder and motor developmental delay at a median age of 6 months (range 2–9 months). All reported a preceding normal perinatal history, birth weight and head circumference. There was no history of a movement disorder, Parkinson’s disease or psychiatric symptoms in either parents or siblings. On review, there was also an associated early history of irritability and feeding difficulties from the neonatal period to age 3 months in all these patients.

Of particular interest was the clinical progress of Case 4 who is the oldest surviving adult patient with classical DTDS—infantile parkinsonism dystonia (now aged 34 years). She presented at 3 months with feeding difficulties and irritability and progressed to develop dystonia and rigidity in infancy associated with motor delay. During early childhood she was able to mobilize with a Kay-walker and had functional use of her upper limbs to steer her electric wheelchair. During adolescence, her movement disorder evolved to progressive bradykinesia and currently at 34 years, she has an asymmetric resting tremor with bradykinesia and minimal purposeful movements in her upper limbs with akinetic lower limbs, muscle wasting and osteopenia. She is hypomimic and non-verbal, requiring gastrointestinal tube feeding. In contrast, her younger sister (Case 5) presented with an early history of feeding difficulties and irritability, then developed a progressive hyperkinetic dystonic phenotype (without parkinsonian features) and died at age 10 years from pneumonia. Both siblings were only recently genetically diagnosed with DTDS in 2010, after discovery of the disease-causing gene. Indeed before diagnosis, Case 4 had a diagnostic label of ‘juvenile parkinsonism’.

Case 6 represents the most classical clinical phenotype (Kurian *et al.*, 2011*a*) with initial infantile hyperkinesia (dyskinesia/dystonia) and axial hypotonia, progressing in childhood to a parkinsonian bradykinetic movement disorder (Supplementary Video 4; section 1). Three children were noted to have axial hypotonia at presentation and with time all five cases developed a movement disorder characterized by axial hypotonia, hypomimia, ridigity, generalized bradykinesia, generalized dystonia with dystonic tremor (Supplementary Video 4; section 2). Two children developed chorea of their lower limbs and had paroxysmal dystonic storms. Two cases had eye movement abnormalities with saccadic intrusion on smooth pursuit in one and recurrent oculogyric crises in the other. These two were also noted to have orolingual dyskinesia as well as dystonic tremor of tongue and limbs. All patients developed bulbar dysfunction and required artificial feeding through the nasogastric/gastrostomy route.

Cases 4 and 6–8 underwent neurotransmitter analysis and all found to have raised CSF HVA: HIAA ratio in keeping with DTDS (median ratio 7.2, range 6.8–31.9, and normal ratio range 1.0–4.0, Table 2). MRI brain imaging was normal in all those who had imaging for Cases 4–8 and included standard axial and coronal T_1_, T_2_ sequences with FLAIR and diffusion-weighted imaging.

**Table 2:** CSF neurotransmitter profiles and results of magnetic resonance brain imaging

Case	Age at lumbar puncture	CSF HVA nmol/l (age-related normal limits)	CSF HIAA nmol/l (age-related normal limits)	HVA:HIAA ratio (normal range 1–3.7)	MRI brain
4	34 years	584 (145–342)	86 (67–140)	6.8	Normal
5	–	–	–	–	Normal
6	7 months	1705 (238–867)	218 (114–336)	7.8	Normal
	1 year 2 months	10075 (238–867)	319 (114–336)	31.9	
	4 years 5 months	1342 (231–840)	199 (68–220)	7.1	
	10 months	1271 (294–1115)	185 (129–520)	6.8	Normal
8	9 months	3523 (295–932)	632 (114–336)	6.8	Normal

HVA = homovanillic acid; HIAA = hydroxyindoleacetic acid.

Medical treatments tried in Cases 4–8 included l-DOPA, other dopaminergic drugs, anticholinergics, gamma-aminobutyric acidergic agents and dietary tyrosine restriction. Case 8 was noted to have a minimal (but unsustained) response to l-DOPA with improvement in her irritability and dyskinesia and also showed some marginal temporary improvement in her bradykinesia with selegiline therapy. Overall this group of patients with classical DTDS experience a progressive parkinsonism dystonia movement disorder that is medically refractory.

### *SLC6A3* gene mutational analysis

All eight patients in this cohort were found to harbour either homozygous or compound heterozygous missense or splice site mutations in the *SLC6A3* gene (Table 3). In Case 3, whole exome sequencing data were interrogated for candidate genes causing parkinsonism dystonia, thereby revealing a homozygous change (c.941C>T; Ala314Val) in *SLC6A3.* Retrospective single nucleotide polymorphism (SNP) array analysis confirmed that this *SLC6A3* variant was contained within a 1.4 Mb region of homozygosity shared by the three affected siblings (Cases 1–3), with a different haplotype combination evident in both parents and the unaffected sisters (Fig. 1).

**Table 3:** *SLC6A3* mutations identified in new patients with DTDS

Case	Family	Ethnic origin	Parental consanguinity	Mutation status	Location of mutation and mutation type	Mutations in DNA	Predicted effect on protein
1	1	Pakistani Asian	Yes	Homozygous	Exon 7, missense mutation	c.941C>T	Ala314Val
2	1	Pakistani Asian	Yes	Homozygous	Exon 7, missense mutation	c.941C>T	Ala314Val
3	1	Pakistani Asian	Yes	Homozygous	Exon 7, missense mutation	c.941C>T	Ala314Val
4	2	Mixed European	Yes	Homozygous	Intron 9, splice site mutation	c.1269 + 1G>A	Not known
5	2	Mixed European	Yes	Homozygous	Intron 9, splice site mutation	c.1269 + 1G>A	Not known
6	3	Italian	No	Compound heterozygous	Intron 3, splice site mutation	c.287-5_287-2delinsAAC c.1156G>A	Not known Gly386Arg
					Exon 8, missense mutation		
7	4	Mixed European	Yes	Homozygous	Exon 3, missense mutation	c.1408_1409delinsAG	Tyr470Ser
8	5	Mixed Jewish Ashkenazi/ Iranian/Yemen/ Turkish	No	Compound heterozygous	Exon 2, missense mutation	c.254G>T c.1333C>T	Arg85Leu Arg445Cys
					Exon 10, missense mutation		

For Cases 4–8, mutations were identified by direct Sanger sequencing of *SLC6A3*. The mutations in Cases 4 and 5 were briefly reported previously (Puffenberger *et al.*, 2012). Novel previously unreported mutations were identified in all others. The interval from presentation to definitive genetic diagnosis was markedly delayed (median 8 years, range 0.5–18). Within every patient’s family, identified mutations segregated appropriately with disease status, with parents being heterozygote carriers and siblings being either wild-type or carriers. None of the familial heterozygote carriers (parents or siblings) had symptoms of a movement disorder or psychiatric disease. None of the identified mutations were reported polymorphisms in genomic databases including the 1000 Genomes project (www.1000genomes.org/), dbSNP137 and the NHLBI Exome Sequencing project. Sequence alignment for novel missense mutations showed Ala314, Gly386, Tyr470, Arg85 and Arg445 amino acid residues to be highly conserved throughout mammalian species. The splice site mutations were predicted to cause deleterious splicing leading to either nonsense-mediated decay or a truncated protein product (Berkeley Drosophila Genome project’s splice site prediction, www.fruitfly.org/seq_tools/splice.html).

### *In vitro* heterologous expression system for dopamine transport assay and cell surface expression studies

For all identified missense mutations (atypical DTDS presentation mutant protein = Ala314Val; classical DTDS presentation mutant proteins = Arg85Leu, Tyr470Ser, Arg85Leu, Arg445Cys, co-expressed Arg445Cys–Arg85Leu), the transporter activity of mutant human DAT proteins was compared to that of wild-type human DAT after transient expression of mutant human DAT in LLC-PK1 cells (Table 4). Wild-type human DAT had normal transport activity and amongst all human DAT mutants, Ala314Val (atypical DTDS) displayed the highest ^3^H-dopamine uptake at 8.83 + 0.71% of wild-type and this enabled us to monitor its uptake characteristics in more detail revealing unchanged K_m_, but significantly reduced V_max_ (to 9.9 ± 0.6% of wild-type, mean ± SE, *n = *4, *P = *0.007, Student’s *t*-test). All other mutant proteins showed absent transporter activity (Gly386Arg, Tyr470Ser) or severely reduced activity at ∼0.5–5.65% of normal (Arg85Leu, Arg445Cys) compared with wild-type human DAT. Activity was not further lowered in co-expressed Arg445Cys–Arg85Leu (1.82%).

**Table 4:** Dopamine transport and cocaine analogue binding by wild-type and mutant human DAT

Case	DTDS Type	Human DAT mutant	^3^H-dopamine uptake	^3^H-CFT binding		
				K_d_, (nM)	B_max_, (pmol/mg)	Inhibition by dopamine, K_i_ (µM)
		Wild-type	Control^a^	14.1 ± 2.6	3.47 ± 0.66	4.22 ± 0.44
1–3	Atypical	Ala314Val	8.83 ± 0.71% of WT^c^	5.31 ± 1.47 ^d^	0.24 ± 0.03 ^d^	1.10 ± 0.19 ^d^
6	Classical	Gly386Arg	0 ^b^	5.78 ± 1.42	0.55 ± 0.15 ^d^	8.59 ± 1.13
7	Classical	Tyr470Ser	0 ^b^	34.4 ± 8.8	0.41 ± 0.12 ^d^	19.2 ± 4.9 ^d^
8	Classical	Arg445Cys	5.65 ± 0.18% of WT^c^	128 ± 25 ^d^	2.93 ± 0.54	382 ± 189 ^d^
		Arg85Leu	0.50 ± 0.06% of WT^c^	20.8 ± 7.1	0.65 ± 0.12 ^d^	1574 ± 364 ^d^
		Arg445Cys with Arg85Leu	1.82 ± 0.19% of WT^c^	90.1 ± 19.1 ^d^	2.06 ± 0.40 ^d^	307 ± 97 ^d^

Each case was assessed for transporter properties with corresponding wild-type (WT) controls.

Cases 4 and 5 harboured intronic mutations and experiments were only undertaken in missense mutations identified in Cases 1–3,6,7 and 8 and thus there were four sets of experiments.

Wild-type values were combined in the overall statistical analysis of ^3^H-CFT binding data. All results are for transiently transfected cells. Values shown are mean ± SE.

^a^Uptake was measured with 10 nM ^3^H-dopamine in the assay. Control ^3^H-dopamine uptake activity was ∼0.15 pmol/mg protein/min for the four sets of experiments.

^b^No specific transporter activity (*n = *3).

^c^*P < *0.05 compared with wild-type (one-sample Student’s *t*-test, with wild-type in the set at 100%, Bonferroni-corrected for multiple comparisons; *n = *3 for each expression condition).

^d^*P < *0.05 compared with wild-type (one-way ANOVA followed by Dunnett multiple comparisons with wild-type; *n* = 9 for wild-type and *n* = 3–4 for each mutant expression condition).

Compared with the K_d_ of ^3^H-CFT binding in wild-type human DAT (14.1 nM), the K_d_ was decreased for Ala314Val (5.31 nM) and increased (i.e. reduced affinity) in the mutants Arg445Cys and co-expressed Arg445Cys–Arg85Leu (90.1–128 nM). The values for Gly386Arg, Tyr470Ser and Arg85Leu (5.78–34.4 nM) were not statistically different from the wild-type K_d_.

The K_i_ of dopamine inhibiting cocaine-analogue binding was decreased for Ala314Val (1.1 µM) but increased (i.e. reduced affinity) in most other mutants compared with that of wild-type human DAT with the most marked affinity reduction in Arg85Leu (K_i_ = 1574 μM versus 4.22 µM in wild-type human DAT). Again, co-expression of Arg445Cys–Arg85Leu did not lead to further lowered dopamine affinity.

Maximal binding of ^3^H-CFT to cells mainly represent surface binding (Chen *et al.*, 2004) and this was reduced up to 14 times compared with wild-type in all current human DAT mutants; again, it was not further reduced upon co-expression of Arg445Cys–Arg85Leu.

**Figure 2:**
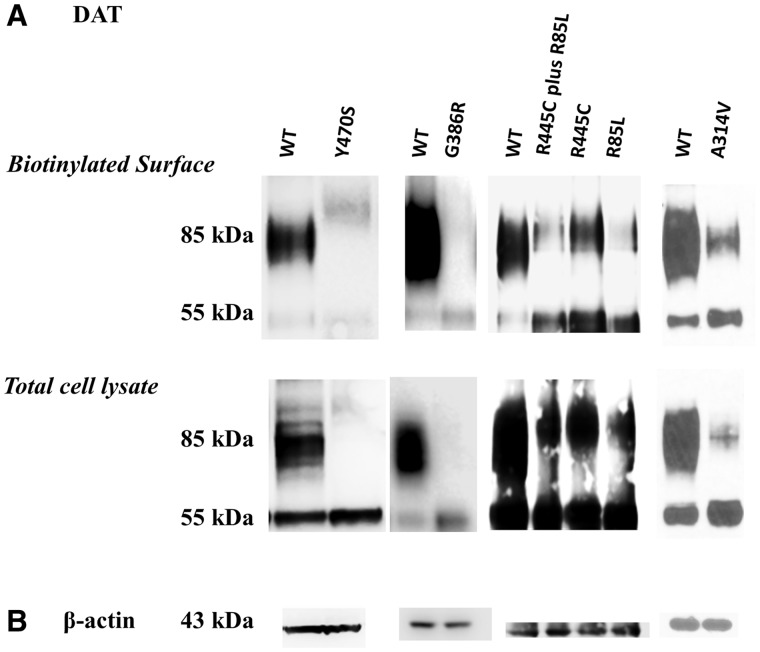
Immunoblotting studies of mutant human DAT. LLC-PK1 cells transient transfected with indicated human DAT constructs were subjected to cell surface biotinylation. Biotinylated proteins (**A**, *top*) and whole cell lysates (**A**, *bottom*) were analysed by western blotting with antibodies against DAT (**A**) and β-actin (**B**). Equal amounts of total lysate protein were loaded for each mutated human DAT as for wild-type examined in the same experiment. There were four sets of experiments with their own wild-type (WT) controls, performed at different points in time, on Tyr470Ser, Gly386Arg, Arg445Cys/Arg85Leu/Arg445Cys-Arg85Leu, and Ala314Val.

Analysis of whole cell lysates by immunoblotting with anti-C-terminal DAT antibody (Fig. 2A bottom portion) showed complete absence of mature glycosylated DAT (85 kDa) in mutants Gly386Arg and Tyr470Ser. There was appreciably reduced expression of mature (glycosylated) DAT for mutants Ala314Val, Arg85Leu, and co-expressed Arg445Cys–Arg85Leu. Mutant Arg445Cys (classical DTDS) showed most expression of mature DAT being only slightly reduced compared with wild-type. In all mutants, the ratio of mature to immature/unglycosylated DAT (55 kDa) had shifted towards the immature species with almost equivalent amounts of unglycosolated DAT compared with wild-type observed in mutants Gly386Arg and Tyr470Ser (with associated absence of glycosolated DAT in these). The biotinylation experiments (Fig. 2A) revealed essentially the same expression pattern.

### Amperometry

**Figure 3:**
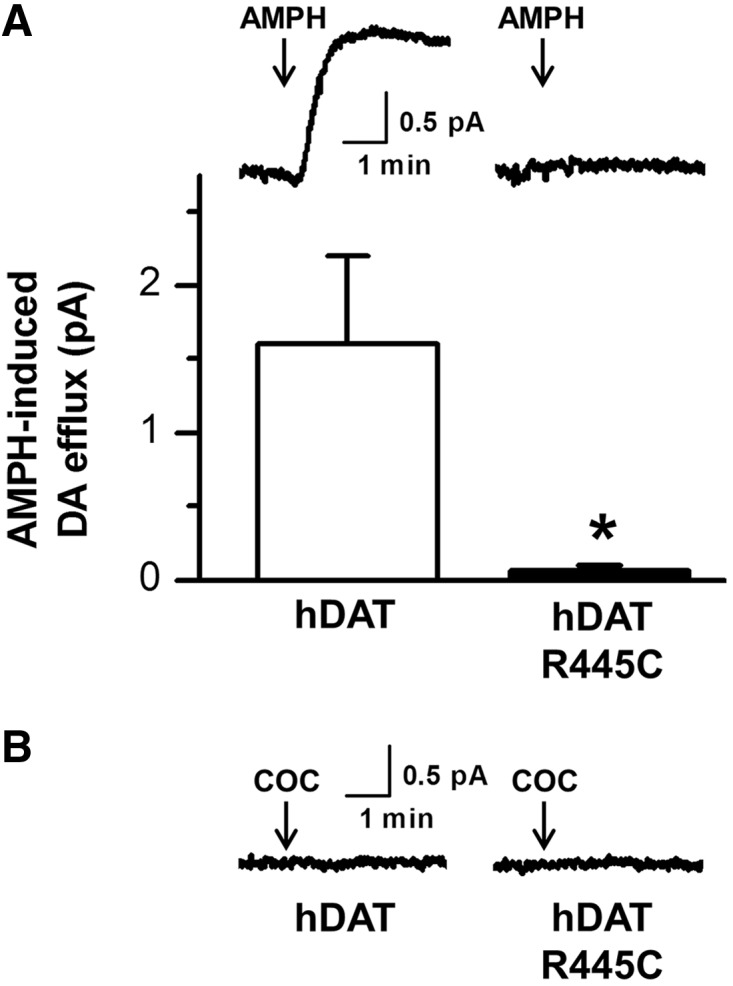
Amperometry studies for human DAT variant Arg445Cys. (**A**) HEK293 cells expressing human DAT or human DATArg445Cys were loaded with dopamine (2 mM) by a whole-cell patch clamp pipette. Amphetamine (10 µM) induces a robust dopamine release from human DAT cells (quantified by amperometric current). Amphetamine fails to induce dopamine release from human DAT Arg445Cys cells (*P < 0.05 Student’s *t*-test human DAT versus human DAT Arg445Cys, *n = *4). (**B**) The DAT inhibitor cocaine (COC; 10 µM) does not alter the amperometric current signal in human DAT nor human DAT Arg445Cys (*n = *3), indicating that there is not a constitutive DAT-mediated dopamine efflux.

Amperometry studies were possible for mutant Arg445Cys as there was appreciable expression on the cell surface, unlike other mutants. Cells expressing wild-type and mutant human DAT Arg445Cys were loaded with dopamine (2 mM) by a whole-cell patch clamp pipette. Amphetamine (10 µM) induced a robust dopamine release from wild-type human DAT cells (quantified by amperometric current). Amphetamine failed to induce dopamine release from mutant human DAT Arg445Cys cells (*P < 0.05 Student’s *t*-test wild-type human DAT versus human DATArg445Cys, *n = *4). The DAT inhibitor cocaine (10 µM) did not alter the amperometric current signal in human DAT nor human DATArg445Cys (*n = *3), indicating that there was no constitutive DAT-mediated dopamine efflux (Fig. 3).

## Discussion

DTDS is a recently described neurotransmitter disease that is classically characterized by an infantile onset, complex progressive motor disorder that is pharmacoresistant and life-limiting (Kurian *et al.*, 2009, 2011*a*). We now present the clinical, biochemical and genetic features of a cohort of newly identified DTDS patients including atypical disease presentation in adolescents and the first patient with infantile onset DTDS surviving into adulthood. We propose that mutations in *SLC6A3* not only cause disease in infancy, but can also result in a phenotypic spectrum of severe progressive movement disorders with onset in childhood/adolescence and also in adulthood (Henriksen *et al.*, 2012) that has not been previously reported. We propose that DTDS should now be recognized as a phenotypic continuum with variable age of onset from infancy (classical DTDS, disease onset <1 year), atypical juvenile and adult onset DTDS (Henriksen *et al.*, 2012).

Our cases highlight the presentation of adolescents who have DTDS associated with *SLC6A3* mutations, with later disease presentation and a case of classical infantile DTDS patients surviving into adulthood. We describe the clinical course of three brothers presenting in early adolescence with tremor with severe disease progression leading to a ‘juvenile parkinsonism’ phenotype with onset of hypomimia, speech difficulties and then frank parkinsonism with hand tremor, bradykinesia and ocular flutter over two decades. Our finding of this phenotypic spectrum and later disease onset is further corroborated by an abstract documenting a 40-years-old patient with early-onset Parkinson’s disease presenting with unilateral hand tremor at 28 years, associated with abnormal DAT scan imaging. This patient with clinical features of early-onset Parkinson’s disease was found to be compound heterozygous for *SLC6A3* mutations (Ile312Phe, Asp421Asn) (Henriksen *et al.*, 2012).

In Family 1 with atypical DTDS, unusually all male children are affected whereas the five sisters are unaffected. Overall in DTDS there does not appear to be a clear specific gender bias, as our previously reported cohort of infantile DTDS (Kurian *et al.*, 2011*a*) included nine females and two males. Review of all current cases of DTDS suggests a slight female preponderance of 1.5 females: 1 male (data collated from current manuscript; Kurian *et al.*, 2011*a*; Henriksen *et al.*, 2012), but it is not possible to consider a meaningful gender effect in DTDS with such limited sample size.

The eye movement abnormalities of DTDS include oculogyric crises, ocular flutter and saccade initiation failure (Kurian *et al.*, 2011*a*). Oculogyric crises are also characteristically seen in other monoaminergic neurotransmitter disorders associated with dopamine deficiency, including tyrosine hydroxylase deficiency, alpha aromatic decarboxylase deficiency and pterin defects (Willemsen *et al.*, 2010; Kurian *et al.*, 2011*b*) and also as drug-induced-phenomena (Darling *et al.*, 2013). Although many ophthalmic symptoms are observed in other extrapyramidal movement disorders (including Parkinson’s disease-associated dry eye, decreased blink rate, vergence dysfunction, progressive supranuclear palsy-related lid retraction, frequent square-wave jerks and supranuclear palsy) (Clark and Eggenberger, 2012), oculogyric crises and flutter are rarely reported and therefore may aid in the clinical discrimination of patients with DTDS with atypical late-onset disease from other adult parkinsonian syndromes or other monogenic causes of Parkinson's disease.

In this report, we additionally describe the first patient with infantile onset DTDS surviving into adulthood who was only genetically confirmed at 34 years of age. At this stage in her 30 s, she had features of parkinsonism with prominent bradykinesia, and was clinically labelled as having ‘juvenile parkinsonism’. Although she showed many of the early features of classical infantile DTDS (including early childhood dystonia, pharmacoresistance to therapeutic agents and eventual clinical progression to akinesia) (Kurian *et al.*, 2009, 2011*a*) her relative longevity into adulthood suggests that there may be a subgroup of adults with ‘juvenile Parkinson’s disease’ who have undiagnosed DTDS, in whom either the early disease course was not recognized or CSF neurotransmitter analysis has not been undertaken. Interestingly, the sister of the patient described above (with the same homozygous *SLC6A3* mutation), presented differently, with a hyperkinetic dystonic movement disorder who died at age 10 years. We postulate that such intrafamilial variation in clinical disease severity and presentation may be due to other currently undetermined genetic influences and/or environmental factors.

**Figure 4:**
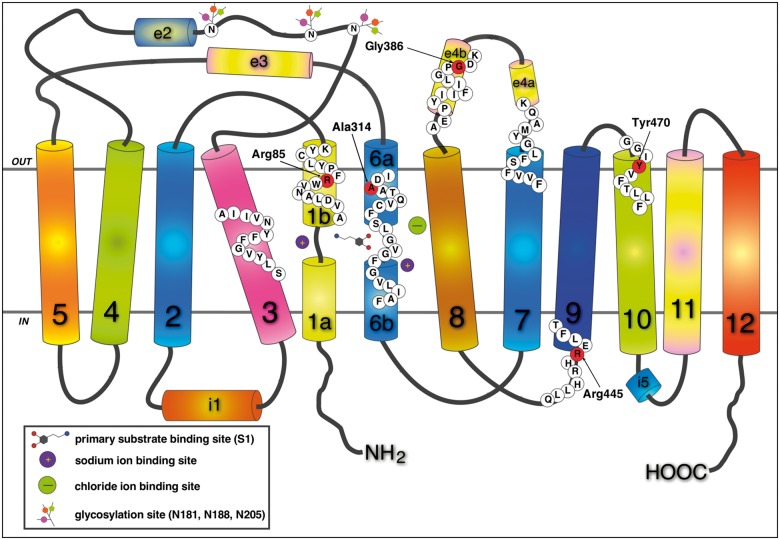
Schematic representation of the dopamine transporter. The DAT consists of 12 transmembrane domains (TM1–12) connected by extracellular (EC) and intracellular (IC) loops, some of which include helical portions (e2, e3, e4a, e4b, and i1, i5, respectively). Amino acids are indicated by white circles and mutated amino acids by red circles. All amino acids subject to missense mutation are identified: Arg85 (R) is shown at transmembrane domain 1 b, Gly386 (G), in the e4b portion of EC loop 4, Ala314 (A) at domain 6 a, Arg445 (R) at the bottom of TM9 and Try470 (Y) at the top of TM10.

DAT consists of 12 transmembrane protein domains (TM1–12; Fig. 4) functioning as a gated channel with two conformations either open exclusively to the extracellular or intracellular milieu (Yamashita *et al.*, 2005). These are delineated as outward-facing and inward-facing, respectively, and when the extracellular substrate binds to its primary binding site termed S1, alongside Na^+^ and Cl^−^, this results in a conformational change from outward-facing to inward-facing (Yamashita *et al.*, 2005; Kniazeff *et al.*, 2008; Shan *et al.*, 2011). In the bacterial leucine transporter LeuT molecular model (Yamashita *et al.*, 2005; Singh *et al.*, 2007; Zhou *et al.*, 2007) the conformational change mechanism is a result of continuous interruption and reformation of a salt bridge between Arg60 in the N-terminus close to the cytoplasmic end of TM1 and Asp346 at TM8; this salt bridge is stabilized by a cation-π interaction between Arg60 and Tyr335 at the cytoplasmic end of TM6 (Kniazeff *et al.*, 2008). Another binding site termed S2 is located at the inner end of the extracellular cavity of the transporter, just above the extracellular gate, which (when closed) includes a salt bridge between Arg85 in TM1 and Asp476 at the top of TM10 (Torres *et al.*, 2003*b*; Kniazeff *et al.*, 2008; Shan *et al.*, 2011). The S2 site is considered to be relevant in the overall substrate translocation cycle (Beuming *et al.*, 2006; Shi *et al.*, 2008).

**Figure 5:**
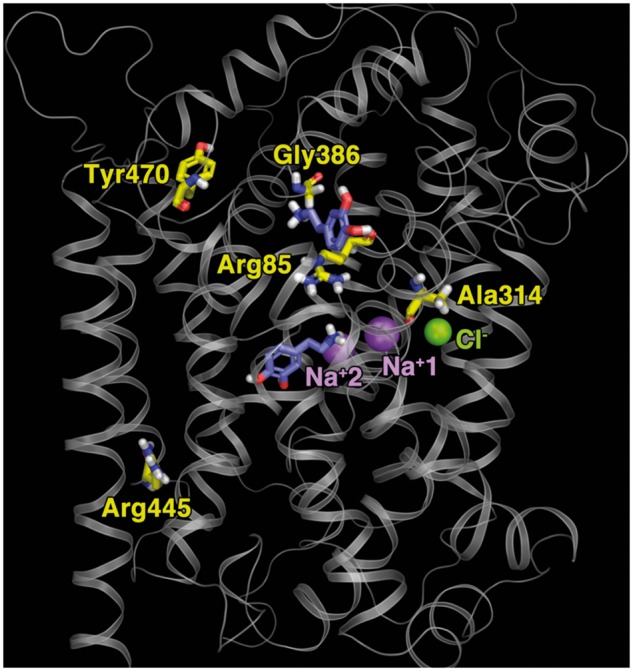
Structural homology modelling of DAT based on LeuT. Transmembrane domains and loops are in grey; mutated amino acids affected by missense mutations in yellow and bound ions in purple (Na^+^) and green (Cl^−^).

The Ala314Val (atypical juvenile DTDS) is an intriguing mutant and distinct among the mutants studied here, retaining the highest residual dopamine uptake capability [8.8% for uptake measured at a dopamine concentration far below its K_m_ (Table 4) and 10% as inferred from a 90% drop in V_max_ with unchanged K_m_, data not shown]. This may, in part, underlie the later-onset juvenile parkinsonism associated with this mutation in comparison to the mutations identified in classical DTDS. Ala314 is the direct neighbour of Asp313, an S2 residue in human DAT studied by our group previously (Chen *et al.*, 2004). In our LeuT-based homology model (Fig. 5), the aliphatic side-chain of Ala314 is pointing outward with respect to the S2 pocket, and thus its increased bulk when replaced by valine can be expected to have less impact on DAT function than the other DAT-mutants identified in the classical DTDS with infantile parkinsonism dystonia phenotype. For example, the mutation of Arg85Leu (classical DTDS) is part of the extracellular gate and this point mutation inhibits normal gate closure during transport, and therefore would be expected to interfere with DAT function more severely than Ala314Val. The Tyr470 (classical DTDS) is just outside S2 (Fig. 5), at the beginning of TM10 at the extracellular side; it is conceivable that its mutation conformationally affects nearby residues identified as part of the extracellular permeation pathway (Shan *et al.*, 2011). In addition the Gly386 (classical DTDS, Fig. 5) is located in extracellular loop 4 (EL4), a region with great conformational mobility and likely to be involved in the cycle for substrate translocation (Shan *et al.*, 2011). The corresponding Ala319 in LeuT also changes position with antidepressant binding to LeuT (Singh *et al.*, 2007). Our homology modelling of DAT based on LeuT shows EL4 as a hairpin folding back into the membrane interior, with arginine in the 386 position pointing its side chain with steric bulk into the S2 binding site, thereby obscuring it and likely interfering with substrate translocation.

The Arg445 is located in the lower portion of TM9 and Arg445Cys (classical DTDS, Fig. 5) effects on DAT structure-function relationships are less predictably deleterious and appears distinct to the other mutations identified. Our immunoblot studies for Arg445Cys human DAT show appreciable levels of mature DAT compared with wild-type, suggesting that for this mutant, unlike the majority of other mutants, there is both near-normal DAT glycosylation and trafficking of the mature transporter to the cell surface. Despite this, it is clear that Arg445Cys does indeed negatively impact DAT function with impaired dopamine uptake, dopamine recognition and binding (Table 4). In addition we have demonstrated the striking loss of amphetamine-mediated dopamine efflux for Arg445Cys, further indicating this mutation is likely to severely impact on general DAT conformational capability, in either inward or outward flux direction. It is understood that amphetamine (as is dopamine) is taken up by DAT and thus, reduced substrate uptake capability may limit intracellular amphetamine accumulation and therefore amphetamine-induced dopamine efflux. However, Arg445Cys does maintain some substrate transport capacity [amphetamine was applied at a concentration (10 µM) much higher than K_m_ for human DAT], amphetamine uptake, and the hydrophobicity of amphetamine likely permits some translocation across the plasma at high concentrations (Sulzer *et al.*, 2005). The total lack of dopamine efflux even with 2 mM dopamine directly perfused into the cytoplasm is striking and suggests a direct effect of Arg445Cys on the reverse transport capability of DAT.

Our present studies on mutant human DAT demonstrate impaired transporter function is likely to be multifactorial and involves: (i) loss of the primary function of DAT as indicated by absent or severely reduced dopamine uptake; (ii) reduced DAT cell surface binding; (iii) in most cases reduced affinity of DAT for dopamine; (iv) generally reduced dopamine recognition by DAT; and (v) decreased expression of mature DAT with an predominance of excess unglycosylated DAT, which is known to negatively impact both the transport function of DAT and also trafficking of DAT to the cell surface (Torres *et al.*, 2003*b*; Torres, 2006). Our data highlight that further work is required to determine the exact mechanisms by which mutant human DAT, and Arg445Cys in particular, lead to disease and the newly identified DTDS clinical phenotypes, such as further investigation of undetermined effects of structure, aberrant DAT folding and oligomerization defects. In our previous work on DTDS, the postulated disease mechanism is loss of dopamine re-uptake, which leads to depleted presynaptic dopamine stores and excessive extraneuronal dopamine, which may also overstimulate presynaptic D2 autoreceptors leading to inhibition of tyrosine hydroxylase and subsequently reduce dopamine production (Blackstone, 2011). Excess extraneuronal dopamine may also cause further consequences such as desensitization of postsynaptic dopamine receptors (with alterations in downstream signalling) and dopamine-induced genotoxicity (Gainetdinov and Caron, 2003; Stopper *et al.*, 2009).

As reported in this study and by our group previously, the majority of infantile cases the mutant human DAT retain 0–5% residual DAT activity whereas the human DAT mutants Ala314Val (atypical juvenile DTDS cases) retain higher residual dopamine. Furthermore human DAT mutants of atypical juvenile DTDS cases demonstrate some residual and even up to normal levels of expression of mature transporter protein at the cell surface (Henriksen *et al.*, 2012), whereas surface expression of mature DAT in classical infantile-onset DTDS is severely reduced or absent (Kurian *et al.*, 2009, 2011*a*). These atypical juvenile and adult DTDS (Henriksen *et al.*, 2012) cases provide insight into potential genotype–phenotype correlations in DTDS and suggest that different *SLC6A3* genotypes may therefore have a differential impact on DAT dysfunction. Our eldest atypical DTDS patient at age 28 years appears to have a more severe disease progression than that of the patient described by Henriksen *et al.* (2012) and this may be explained by differing DAT residual activity. Our findings in combination, would suggest that residual DAT activity may result in postponing DTDS disease onset from infancy to adolescence or even adulthood. It is also likely that other currently undetermined genetic and environmental factors may play a role in onset and severity of disease.

Our study suggests that mutations in *SLC6A3* lead to a continuum of DTDS phenotypes whereby the clinical phenotype appears to be related to residual DAT function, which determines the onset and severity of symptoms. Higher residual DAT activity may postpone the age of disease onset. Similar phenotypic continuums (influenced by the level of residual protein function) are observed in other neurotransmitter disorders such as tyrosine hydroxylase deficiency (Willemsen *et al.*, 2010; Pons *et al.*, 2013).

The differential diagnosis for genetic dystonia parkinsonism continues to grow with an ever expanding number of causes and syndromes identified in both infants/children (neurotransmitter defects, metal storage diseases, mitochondrial disorders, lysosomal storage disorders) (García-Cazorla *et al.*, 2011) and adolescents/adults (parkin, *DJ1* and *PINK1*-related Parkinson’s disease, *PLA2G6-*associated neurodegeneration, Kufor-Rakeb disease and beta-propeller protein-associated neurodegeneration syndrome) (Schneider and Bhatia, 2010; Haack *et al.*, 2012). In addition to these disorders, we propose that DTDS should now also be a differential diagnosis for juvenile Parkinson’s disease. We advocate that in adolescents or adults with a clinical picture of juvenile parkinsonism (onset <20 years) investigations for DTDS should be considered, including: (i) CSF neurotransmitter studies; (ii) DAT SPECT scan (abnormal in DTDS) (Kurian *et al.*, 2011*a*); and (iii) *SLC6A3* sequencing*.* Such investigations should assist differentiation of juvenile parkinsonism as a result of DTDS from other parkinsonian disorders.

In conclusion, we report a cohort of patients with DTDS identified in infants and adolescents and provide data corroborating loss of DAT function in this condition. DTDS is now not only described in children with infantile-onset parkinsonism dystonia, but also in adults with juvenile-onset parkinsonism and a new case report of early onset Parkinson’s disease (Henriksen *et al.*, 2012). DTDS can mimic a number of movement disorders and should therefore be investigated in atypical dystonic/dyskinetic/spastic cerebral palsy, progressive childhood movement disorders, juvenile parkinsonism, parkinsonism dystonia and early onset Parkinson’s disease in adults. Further elucidation of disease mechanisms related to this transportopathy will provide insight into the processes underpinning this progressive disorder, as well as identifying potential therapeutic targets for this pharmacoresistant condition.

## Supplementary Material

Supplementary Data

## Abbreviations

CFT: 2β-carbomethoxy-3β-[4-fluorophenyl]-tropane
DAT: dopamine transporter
DTDS: dopamine transporter deficiency syndrome
