# Supplementary material for: Dopamine transporter deficiency syndrome: phenotypic spectrum from infancy to adulthood
Source: Brain. 2014 Mar 10;137(4):1107–19. doi: 10.1093/brain/awu022 (PMC3959557; doi:10.1093/brain/awu022)
Supplement: Supplementary Data [file supp_137_4_1107__index.html]

Dopamine transporter deficiency syndrome: phenotypic spectrum from infancy to adulthood — Supplementary Data 

# Dopamine transporter deficiency syndrome: phenotypic spectrum from infancy to adulthood

## Supplementary Data

files

**Files in this Data Supplement:**

- Supplementary Data - mp4 file
- Supplementary Data - mp4 file
- Supplementary Data - mp4 file
- Supplementary Data - mp4 file
- Supplementary Data - mp4 file
- Supplementary Data - docx file
