# Supplementary material for: Dopamine transporter deficiency syndrome: phenotypic spectrum from infancy to adulthood
Source: Brain. 2014 Mar 10;137(4):1107–19. doi: 10.1093/brain/awu022 (PMC3959557; doi:10.1093/brain/awu022)
Supplement: Supplementary Data [file supp_awu022_brain-2013-01574-File016.docx]

**VIDEO LEGENDS**

**Video 1: Case 1**

The youngest brother (IV:8) with atypical DTDS age 16 years with head tremor/ titubation. He has preserved facial expression and no hand tremor.

**Video 2: Case 2**

The middle brother (IV:5) with atypical DTDS age 26 years with neck dystonia and hypertrophy of trapezius muscles, frontalis hyperactivity and cervical antecollis. He has subtle intermittent head tremor. He has hypomimic facies with normal eye movements.

**Video 3: Case 3**

The eldest brother (IV:4) with atypical DTDS (at 28 years) indicates hypomimic facies with marked head titubation/tremor and ocular flutter. There is marked coarse bilateral upper limb tremor of varying intensity which is not affected by posture.

**Video 4: Case 6**

**Section 1**: Case 6withclassical DTDS(at 6 months) indicating mild generalised dyskinesia (including mild orolingual dyskinesia), dystonic posturing of upper limbs, clawed dystonic posturing of the feet, severe axial hypotonia, and paucity of spontaneous movement with bradykinesia. There appears to be preservation of facial expression.

**Section 2:**Video demonstrating the typical l progression of classical DTDS in patient 6 (at 4 years) with distal upper limb dyskinesia and tremor, dystonic posturing of fingers, hypomimia and paucity of spontaneous voluntary movement.
